# A Bayesian Hierarchical Model for Relating Multiple SNPs within Multiple Genes to Disease Risk

**DOI:** 10.1155/2013/406217

**Published:** 2013-12-31

**Authors:** Lewei Duan, Duncan C. Thomas

**Affiliations:** Division of Biostatistics, Department of Preventive Medicine, University of Southern California (USC), 2001 N. Soto Street, Los Angeles, CA, USA

## Abstract

A variety of methods have been proposed for studying the association of multiple genes thought to be involved in a common pathway for a particular disease. Here, we present an extension of a Bayesian hierarchical modeling strategy that allows for multiple SNPs within each gene, with external prior information at either the SNP or gene level. The model involves variable selection at the SNP level through latent indicator variables and Bayesian shrinkage at the gene level towards a prior mean vector and covariance matrix that depend on external information. The entire model is fitted using Markov chain Monte Carlo methods. Simulation studies show that the approach is capable of recovering many of the truly causal SNPs and genes, depending upon their frequency and size of their effects. The method is applied to data on 504 SNPs in 38 candidate genes involved in DNA damage response in the WECARE study of second breast cancers in relation to radiotherapy exposure.

## 1. Introduction

The Women's Environment, Cancer And Radiation Epidemiology (WECARE) study [1] is aimed at a comprehensive examination of genes involved in particular functional pathways. The study is a population-based nested case-control study of 708 contralateral breast cancers (CBC) within a notional cohort of about 65,000 survivors of a first breast cancer, 1401 of whom serve as controls, and the primary exposure of interest is ionizing radiation dose to the contralateral breast from radiotherapy for treatment of the first cancer. Ionizing radiation is known to cause double strand breaks (DSBs) in DNA, which can invoke any of several DNA damage response mechanisms, primarily DSB repair via either homologous recombination or nonhomologous end joining, cell cycle checkpoint regulation, or apoptosis. The original study focused on mutations in the *ATM* gene, which plays a central role in the recognition of DSBs. The study was then extended to include *BRCA1*,* BRCA2*, and *CHEK2*, which are all involved in homologous recombination repair (HRR), and later still to include a broader set of 38 candidate genes involved in this and other pathways for DSB damage response. We have previously reported on the main effects of ionizing radiation [2, 3], *ATM* [4–6], *BRCA1/2* [7–12], *CHEK2* [13], and the interactions of radiation with *ATM* [14] and *BRCA1/2* [15] as well as with other treatments and reproductive factors [16, 17], amongst other risk factors. The aim of this paper is to provide a comprehensive modeling strategy for examining the effects of *all* genes in a pathway and to apply the approach to candidate genes for CBC risk in the WECARE study.

There are a growing number of literature works on methods for pathway modeling, motivated in large part by an interest in mining GWAS data for commonalities across related genes that individually may not achieve genomewide significance but in the aggregate may point to novel pathways (see [18] for a review of gene set enrichment analysis and alternatives). Our goal here is more modest, guided by an *a priori *selection of strong candidate genes [19]. Like other methods of pathway analysis, however, we aim to exploit external knowledge about the biological function of each gene and the relationships between them [20].

Our starting point is a model for multiple variants proposed by Quintana et al. [11], which collapses a subset of the variants within a gene into a single “burden” type index, similar to a number of other recent rare variant proposals (see Basu and Pan [21] for a review and comparison by simulation), but extended to allow for both deleterious and protective effects and to explicitly allow for uncertainty about which variants to include in the model (and which direction for those that are included) by Bayesian model averaging. This approach was further extended to incorporate prior covariates in the probabilities of SNP inclusion [12, 22]. Hoffman et al. [23] introduced a step-up variable selection approach that allows for deleterious and protective effects but did not consider model uncertainty except in the form of a permutation procedure for the overall significance test so is unable to assess the importance and direction of particular variants or alternative models. Chen et al. [24] describe a somewhat similar model that combines variable selection at the SNP level with shrinkage at the gene level. In the current paper, we extend this approach to multiple genes, incorporating prior covariates and prior gene-gene similarity information in a hierarchical modeling framework.

## 2. Model Specification

**Figure 1:**
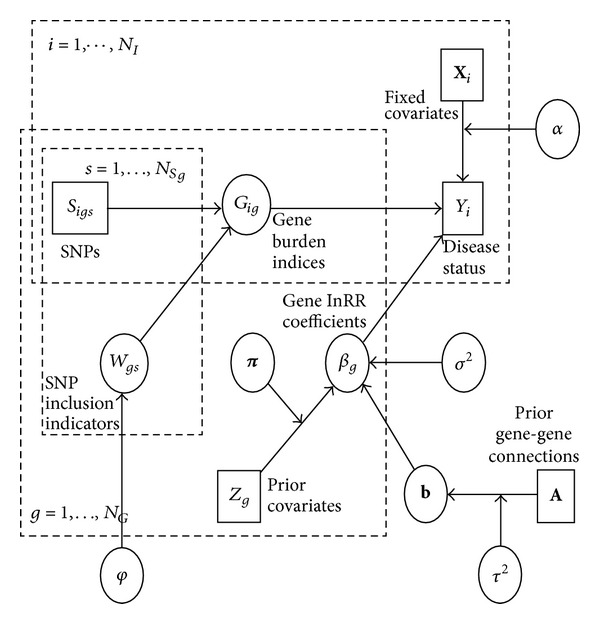
Directed acyclic graph describing the structure of the model. Boxes describe observed data; circles represent latent variables or model parameters. Single arrows denote stochastic relationships, while double arrows denote deterministic relationships. The first rectangle illustrates the relations of disease status and genes at the subject (i) level; the second rectangle illustrated the relations of external information and first level coefficient *β*_*g*_ at the gene (*g*) level; the third rectangle illustrates the relations of weighted SNP effects and gene burden index at SNP (s) level.

We have information on *i* = 1 ⋯ *N*_*I*_ individuals with binary outcomes *Y*_*i*_, a vector of fixed effects **X**_*i*_ (age, family history, etc.), and a vector of SNP genotypes **S**_*ig*_ = (*S*_*igs*_),  *s* = 1 ⋯ *N*_*S*_*g*__ within multiple genes *g* = 1 ⋯ *N*_*G*_ for each individual. We propose a novel model based on a hierarchical Bayes framework with three levels: (i) a subject-level model for the association between genes and disease, (ii) a gene-level model for the regression coefficients in the gene-disease association model, and (iii) a SNP-level model describing which variants contribute to each gene and the direction of their effects. (These submodels are described by (1), (2), (4), and (5), resp., below and the surrounding text.) The general framework is similar to one recently proposed by Quintana et al. [12, 22] but differs in a number of details. The overall model is represented as a directed acyclic graph in Figure 1, where boxes represent observed data and circles represent latent variables or model parameters; single arrows denote stochastic links, while double arrows denote deterministic links. The 3 dotted rectangles enclose the covariates and parameters included in each level of the model and their relations.

The subject-level model is specified in terms of a burden index for each gene, a deterministic function comprised of the number of positively associated SNPs minus the number of negatively associated SNPs; however, the choice of whether a SNP is included or not and, if included, its direction is stochastic, governed by prior probabilities that could in principle vary across genes or across SNPs within genes. The gene-level model has means and covariances for each ln RR (relative risk in log scale) coefficient that can depend upon external information (“prior covariates” and prior “gene-gene connections”). In principle, the SNP-level model could also include prior covariates [22], although that is not considered here. For the simulations and the analysis of the real WECARE data, we used the Gene Ontology (GO, [26] a pathway ontology database, http://www.geneontology.org/) for the 38 WECARE candidate genes to construct the prior covariate and connection information, as described in more detail in the simulation section.

*Level 1*. The subject-level model for case-control data uses a conditional logistic regression model to relate burden indexes *G*_*ig*_ = *G*(**W**_*g*_, **S**_*ig*_) for genes *g* = 1 ⋯ *N*_*G*_ to a binary outcome variable *Y*_*i*_, the disease status for individual *i*. Here, *G* denotes a deterministic function of the SNP genotypes *S*_*igs*_ for SNP *s* in gene *g* with corresponding weights **W**_*g*_ = (*W*_*gs*_) ∈ {−1, 0, + 1} defined in the level 3 model. Thus, the first level model is of the following form:

(1) logit  Pr(Yi=1)=Xi′α+∑g=1NGeβgG(Wg,Sig)+offseti,

where **X**_*i*_ denotes a vector of fixed covariates (confounders) with coefficient vector **α**. The offset term is needed to account for the counter-matched design in the WECARE study [1].

Each gene burden index has a log regression coefficient *e* ^*β*_*g*_^ describing its contribution to risk, the interpretation of which will depend upon the current assignment of weights. A change of the genotype of a single SNP in the function *G*_*ig*_ is reflected by the change of *e* ^*β*_*g*_^ on logit scale. This is based on all SNPs tested in the gene, but each SNP has a different weight *W*_*gs*_ with different prior probabilities; the details are explained in level 3 of the model. The exponentiation of the **β**s ensures that the effects of each gene will be positive, thereby avoiding the label-switching problem that would arise if the signs of *β*_*g*_ and all the *W*_*gs*_ were reversed for a given gene. This also avoids having to deal with truncated normal distributions if *β*_*g*_ were not exponentiated but instead constrained to be positive. (We call (1) Model I and briefly describe this alternative possibility (Model II) in Section 7.)

*Level  2*. The regression coefficients *β*_*g*_ in the first level logistic regression model are given by the gene level of the hierarchical model:

(2) $$\begin{array}{c} \beta_{g}=Z_{g}^{\prime }\pi +b_{g}+e_{g}, \end{array}$$

where

(3) $$\begin{array}{c} \pi =\left(\pi_{0},\ldots ,\pi_{N_{Z}}\right)\sim N\left(0,V_{\pi }I\right),\\ b=\left(b_{1},\ldots b_{N_{G}}\right)\sim N\left(0,\tau^{2}A\right),\\ e\;=\;\left(e_{1},\ldots ,e_{N_{G}}\right)\sim N\left(0,\sigma^{2}I\right). \end{array}$$

The level 2 model uses a simple linear regression to relate the regression coefficients **β** from the level-1 model to external information on the genes' involvement in certain pathways and the similarity of their effects. We incorporate information regarding prior predictions of the effects of each gene into the design matrix **Z**, here structured as a gene-by-pathway matrix of binary values, each indicating whether a gene is in a particular pathway. Basically, **Z** contains second-stage covariates for each of the genetic factors. ***π*** is a column vector of coefficients corresponding to these higher-level effects and is assigned an independent normal prior with mean 0 and variance *V*_*π*_ and identity matrix **I**. Prior information about gene-gene connections is incorporated in the **A** matrix for the **b** random effects with a multivariate normal distribution centered at zero with variance *τ* ^2^. The term **e** is included as a residual error, also given a zero mean independent normal distribution, with *σ* ^2^ specifying the residual variance of the second-stage covariates.

*Level 3*. The SNP-level model defines the deterministic functions *G*(**W**_*g*_, **S**_*ig*_), where each gene is uniquely determined by the SNP inclusion indicator variables *W*_*gs*_. The *G*_*ig*_ serve as a design matrix of genetic factors for the individuals within the study. In other words, the function serves as a risk index for each gene and as a weighted sum of SNP effects within each gene:

(4) $$\begin{array}{c} G\left(W_{g},S_{ig}\right)=\overset{N_{S_{g}}}{\underset{s=1}{\sum }}W_{gs}S_{igs}, \end{array}$$

where the weights *W*_*gs*_ = −1, 0, or +1 have prior probabilities:

(5) $$\begin{array}{c} \text{Pr}\left(W_{gs}=d\right)=\{\begin{array}{cc} \varphi_{-}\frac{\left(\;{\overset{-}{N}}_{s}+c\right)}{\left(N_{S_{g}}+c\right)}, & d=-1\\ 1-\left(\varphi_{-}+\varphi_{+}\right)\frac{\left(\;{\overset{-}{N}}_{s}+c\right)}{\left(N_{S_{g}}+c\right)}, & d=0\\ \varphi_{+}\frac{\left(\;{\overset{-}{N}}_{s}+c\right)}{\left(N_{S_{g}}+c\right)}, & d=1. \end{array} \end{array}$$

Here, *N*_*S*_*g*__ denotes the number of SNPs in gene *g* and ${\overset{-}{N}}_{s}$ the average number of SNPs across all genes; we assigned *c* to be the minimum number of SNPs within any gene. *φ*_+_ and *φ*_−_ represent the parameters of the prior probabilities for deleterious and protective SNP effects, respectively. This form of prior probabilities for the SNP indicator variables keeps the expected number of SNPs included in the model to be roughly similar across genes while allowing genes with more SNPs to have similar probabilities of being included as genes with fewer SNPs. For now, we treat *φ* as fixed parameters, but these too could be given hyperpriors and estimated.

The posterior estimates for the association parameters resulting from the three-level hierarchical Bayesian analysis are an inverse-variance weighted average between the conventional estimates from the logistic regression only and the estimated conditional second-stage means, **Z**_*g*_′***π***. Between the maximum likelihood first-stage estimates and the second-stage prior estimates, the weights will favor the one with smaller variance. This intuitive weight adjustment is one of the important differences between Bayesian hierarchical approach and the single-stage logistic regression analysis.

Finally, the variance components are given standard conjugate inverse gamma hyperprior distributions:

(6) $$\begin{array}{c} \sigma^{2}\sim IG\left({df}_{e},E\right),\\ \tau^{2}\sim IG\left({df}_{b},B\right),\\ V_{\pi }\sim IG\left(1,P\right). \end{array}$$

## 3. Fitting the Model 

The full model is fitted in a sequence of Markov chain Monte Carlo (MCMC) steps described in detail in the Appendix. Basically, the selection of SNPs to include in each gene *W*_*s*_ is performed by sampling from their full conditional distributions one at a time; this involves an approximation to the change in the corresponding estimate of *β*_*g*_ and hence the likelihood that would result from adding or deleting that SNP. The gene-level regression coefficients *β*_*g*_ and correlated random effects *b*_*g*_ are accomplished by the Metropolis-Hastings moves for the entire **β** and **b** vectors, conditional on the current SNPs in the model, the prior covariates **Z**_*g*_, and gene-gene correlation matrix **A**, using a multivariate normal proposal. The second-level gene-level coefficients *π*_*g*_ and the independent and correlated variances *σ* ^2^ and *τ* ^2^ are then sampled using further Metropolis-Hastings moves. Updating the coefficients **α** of the fixed covariates involves only a standard update for logistic regression.

## 4. Posterior Summarization

Instead of parameter estimation, we focus primarily on hypothesis testing and model selection. We use the Bayes factors (BF) at both the SNP level and the gene level to compare the posterior odds provided by data to their prior odds of a pair of hypotheses. Kass and Raftery [27] suggest a qualitative interpretation of BF > 3 (or equivalently 2ln⁡(BF) > 2) as providing “positive” evidence, >20 as “strong” evidence, and >150 as “very strong” evidence.

We tabulate the following quantities, where *D* denotes the ensemble of all the data.

(i) For each SNP, the posterior probability of *W*_*gs*_ = −1, 0, +1 and Bayes factor
  (7) BFgs=(Pr⁡(Wgs≠0 ∣ D)Pr⁡(Wgs=0 ∣ D)) ÷((φ−+φ+)/(NSg+c)1−(φ−+φ+)/(NSg+c)),
 where the first factor is the ratio of posterior probabilities that SNP in gene g has any effect (positive or negative) versus no effect given the data *D* and the second factor is the corresponding ratio of prior probabilities.
(ii) For each gene, the Bayes factor for the probability that at least one SNP is included in the model is
  (8) BFg=(1−Pr⁡(Wg≡0 ∣ D)Pr⁡(Wg≡0 ∣ D)) ÷(1−(1−((φ−+φ+)/(NSg+c)))NSg(1−((φ−+φ+)/(NSg+c)))NSg).
 We also tabulate the posterior means and standard deviations of each, along with the mean number of SNPs included in the model.
(iii) For the other parameters, *α*, **β**, *π*, *σ* ^2^, and *τ* ^2^, we simply tabulate the posterior means and SDs.
(iv) Finally, we tabulate the posterior distributions of numbers of SNPs and numbers of genes with at least one SNP included in the model.

## 5. Simulation Studies

**Figure 2:**
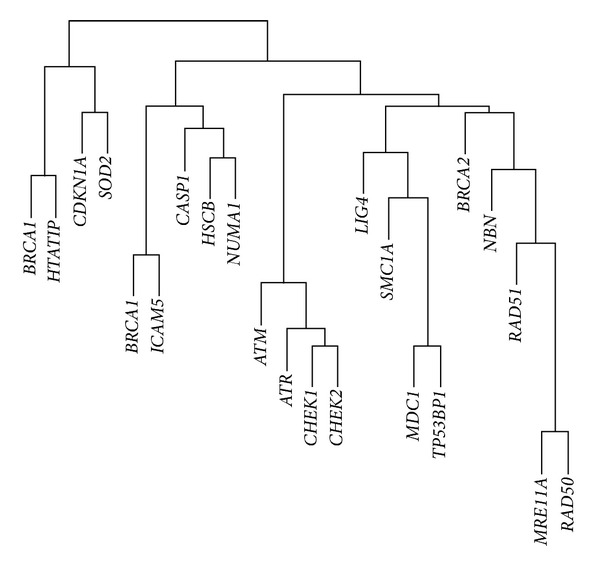
Graphical representation of the A matrix derived from the Gene Ontology. The lower levels of the graph indicate sets of genes with high correlations across the 860 GO terms.

We conducted simulation studies based on the structure of the real WECARE study data described below. Specifically, we used the real SNP, covariate, and counter-matching offset data for each risk set and reassigned case/control status in each risk set based on an assumed relative risk model. We used the estimated values of the coefficients *α* for the fixed covariates and randomly assigned weights *W*_*gs*_ to SNPs and log relative risk coefficients *β*_*g*_ to each gene under the models described above. There were a total of 504 SNPs in 38 genes (ranging from 1 to 51 SNPs per gene) involved in DNA damage response pathways (DNA repair, cell cycle checkpoint control, and apoptosis). Using the Gene Ontology, we extracted 860 terms relating to biological process or molecular function annotated to any of these 38 genes and selected four of these GO terms as prior covariates in the **Z** matrix (specifically, DNA damage checkpoint, MRE11 complex, double-strand break repair via nonhomologous end joining, and negative regulation of cell cycle), with *π* = 0.25,0.5,0.75, and 1 respectively, and the intercept *π*_0_ was set to −2. All 860 GO terms were used to construct a correlation matrix **A** for the similarity in the ways each pair of genes was described in the GO (Figure 2). The log relative risk coefficients *β*_*g*_ were assigned with mean **Z**_*g*_′***π*** and SDs of *b*_*g*_ and *e*_*g*_ *σ* = *τ* = 0.5. SNP weights *W*_*gs*_ were assigned with *φ*_−_ = *φ*_+_ = 0.05 and *c* = 1. The resulting gene indices *G*_*g*_(**W**, **S**) and the corresponding *β*_*g*_, along with the real **X**_*i*_ and estimated **α** coefficients and offset terms, were then used to compute each subject's relative risk and randomly assign which member of each risk set would be designated as the case. The estimates are based on 10 replicates for the data of each of 10 realizations of the *W*_*gs*_ and *β*_*g*_ from these model parameters, using 1000 MCMC scans for tabulation after a burn-in of 500 scans. It yielded a total of 32 causal SNPs in 24 of the genes on average.  Table 1 summarizes the posterior probabilities for SNP and gene inclusion, along with the proportion of SNPs and genes with BFs greater than 3, 20, and 150. Although the differences between null and causal SNPs and genes are somewhat modest, there is a clear shift in both the posterior probabilities and the Bayes factors in the appropriate directions.

## 6. Application to the WECARE Study Data

**Figure 3:**
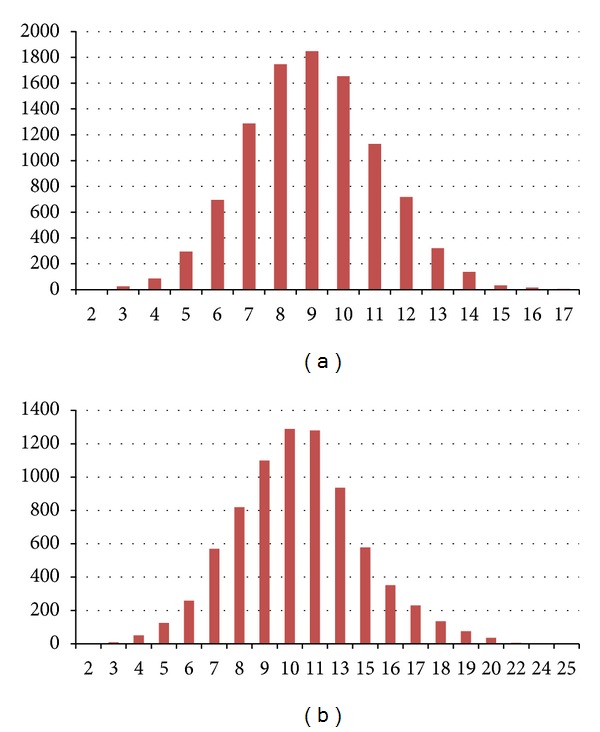
Posterior distributions of numbers of genes (a) and numbers of SNPs (b) included in the analysis of the WECARE study data.

**Figure 4:**
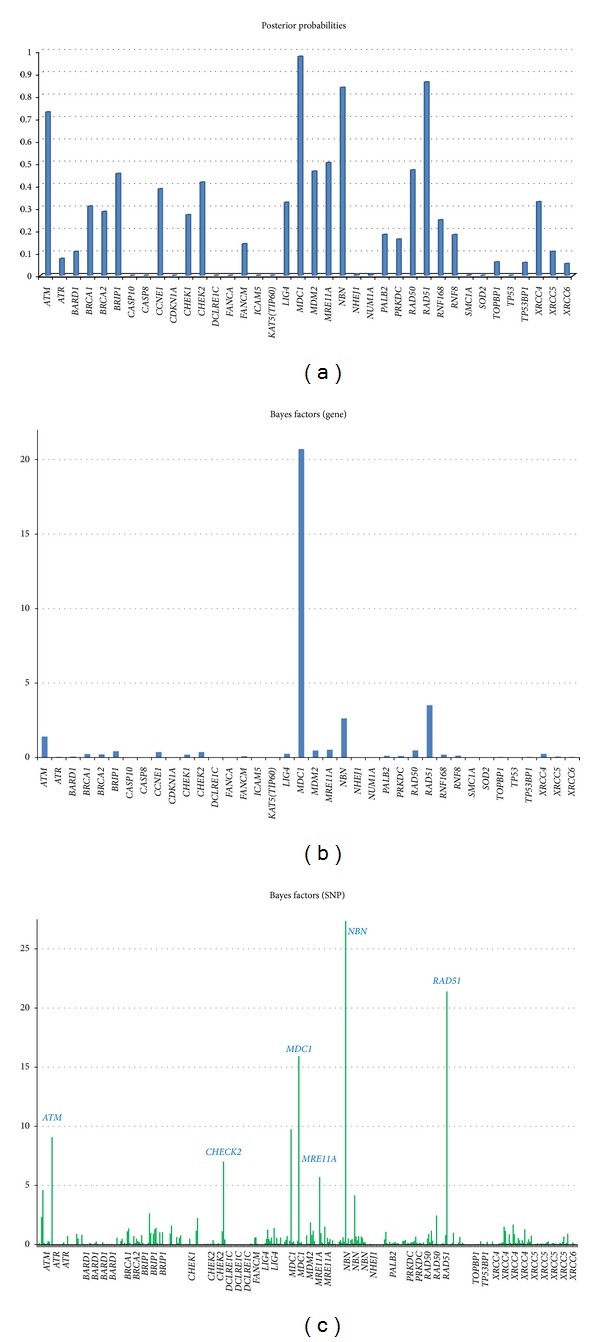
Posterior probabilities (a) and Bayes factors for gene inclusion (b) and SNP inclusion (c) in the model for the real WECARE study data.

Using the same settings as for the simulation studies, we analyzed the real WECARE study data, except that 10,000 scans were retained after a burn-in of 4,000 iterations. The posterior distributions of numbers of genes with at least one SNP included and numbers of SNPs included are shown in Figures 3(a) and 3(b). An average of 10 SNPs in 9 genes was included in the model.  Figure 4 shows the posterior probabilities (a) and Bayes factor for each of the genes (b) and SNPs (c). At the gene level, only *MDC1* and *RAD51 *were included with substantial Bayes Factors of 20.71 (“strong evidence”) and 3.51 (“positive evidence”), respectively, while *ATM* and *NBN* were identified only with BFs between 1 and 3. In this analysis, the known deleterious variants in *ATM*, *BRCA1*, *BRCA2*, and *CHEK2* were treated as fixed covariates rather than being lumped in with the other tag SNPs. None of the four GO terms selected as prior covariates contributed significantly to the model, the strongest being DNA damage checkpoint (*π* = −0.15, SE = 0.27). The correlated variance *τ* ^2^ = 0.25, and the independence variance *σ* ^2^ = 0.16, suggesting moderately strong residual gene-gene similarities (spatiality *τ* ^2^/(*σ* ^2^ + *τ* ^2^) = 61%) defined by the ensemble of all GO terms and not explained by the regression of *β*s on the subset of selected GO terms.

Table 2 lists the numbers of pairs of the homozygous reference allele, heterozygous allele, and homozygous risk allele for cases (CBC) and controls (UBC), respectively, for all the SNPs identified by our models and by a previous WECARE publication [25]. We also report the estimated ln⁡⁡RRs from simple logistic regression for each selected SNP, adjusted for the same set of covariates (age, menarche, menopause, family history, pregnancy, histology, treatment, the *FGFR2* GWAS-identified SNP, and deleterious variants in *ATM*, *BRCA1*, *BRCA2*, *CHECK2s* and offset term) as in our model. The logistic regression found SNPs rs4713354 and rs2269705 in *MDC1 *to be strongly associated with CBC risk (*P* < 0.001), and SNPs rs1800057 v_IVS14 m55, rs13447682, rs3736640, and rs1801320 had protective effects with statistical significance (*P* < 0.05) or with marginal statistical significance (rs6005861 and rs9297757, *P* < 0.1).

Table 2 also shows the SNP Bayes factors, based on which our model selected a total of nine SNPs with positive to strong evidence for disease association. Two SNPs (one in *NBN* and one in *RAD51*) were identified with strong evidence (BF > 20) and seven SNPs from four genes (*ATM*, *CHEK2*, *MDC1*, *MRE11A*) with positive evidence (BF > 3). In a prior study by the WECARE study Collaborative Group, 134 common variants in six DNA damage response genes (*CHEK2*, *MRE11A*, *MDC1*, *NBN*, *RAD50*, and *TP53BP1*) were tested separately or within haplotypes for association with CBC risk [25]. Six SNPs were reported to be associated with CBC risk with *P* < 0.05, but none remained statistically significantly associated after correction for multiple comparisons. Five SNPs (rs6005861 in *CHEK2*, rs4713354 in *MDC1*, rs13447682 in *MRE11A*, and rs9297757 and rs3736640 in *NBN*) among those six SNPs reported by Brooks et al. were selected by our model for showing positive or strong evidence for CBC risk. The remaining SNP (rs14448 in *NBN*) reported by Brooks et al. was not statistically significantly associated with CBC in the logistic regression (*P* = 0.447). All the SNPs except rs4713354 in *MDC1* reported by Brooks et al. were found to have protective effects in the log-additive model. The same direction of the risk was also found for each SNP in the logistic regression. In addition, our model shows positive evidence of CBC risk for SNP rs1800057, a variant in *ATM*, which was previously shown to be associated with a statistically significant reduction in CBC risk [28] in the WECARE study. Its protective effect was also found in the logistic regression (ln RR = −0.47, *P* = 0.046).

Seven of the nine SNPs selected by our model have been found associated with breast cancer risk in previous investigations. Besides the six SNPs reported in the previous WECARE study, rs1801320 (135G > C), a SNP in the 5′-untranslated region (UTR) of the *RAD51* gene, was found with mixed results for its role in breast cancer risk from other breast cancer risk studies [29–31]. In addition to those previously reported SNPs, our model selected rs4987951 in *ATM* and rs2269705 in *MDC1*, about which we found no previous reports of association with breast cancer.

## 7. Discussion

Our model is motivated in part by ongoing work on methods for testing associations with multiple rare variants in next generation sequencing data [12, 22], for which it is obvious that attaining statistically significant results for any single variant is difficult because of their rarity and the enormous multiple comparisons penalty. This motivates our choice of a burden index for gene-level associations comprising simple −1/0/+1 weights with model averaging across their uncertainty distribution. For common variants with minor allele frequencies (MAF) >5% (and perhaps in candidate gene studies for uncommon variants with 1% < MAF < 5%), it may be possible to allow each SNP to have its own regression coefficient from some continuous distribution, but constraints would be needed to ensure identifiability if both SNP- and gene-level parameters were to be estimated.

As a compromise, we have treated the known deleterious variants in *ATM, BRCA1/2*, and *CHEK2 *as fixed covariates, along with age, treatment, reproductive variables, and so forth, since it seems unreasonable to consider these variants as exchangeable with the tagging SNPs. Unfortunately, this precludes borrowing strength across *all* the variants within these genes—that is, given that we know that some variants in these genes are deleterious, it would seem more likely that there would be other causal variants in the same genes. Furthermore, if these four genes have similar prior covariate values **Z**_*g*_, that should inform the estimation of the corresponding *π*_*g*_ *s* and draw the estimates of **β**s for other genes that are highly correlated with them in the **A** matrix towards the *β*_*g*_ values for these genes.

We have included prior information only on genes, not SNPs, in our model, since the GO does not provide any annotation of specific variants within genes. However, there are many ways of classifying SNPs *a priori*, such as simple indicators for whether they are coding or noncoding variants or the predictions of programs like SIFT [32] and PolyPhen [33] based on predicted effects on protein conformation or evolutionary conservation. Such information could easily be incorporated into a multinomial logistic or probit model for the inclusion probabilities *φ*_*s*_ [12, 22]. The current version of our program treats *φ*_+_ and *φ*_−_ as fixed constants, but these could simply be assigned prior Beta distributions, subject to the constraint that *φ*_+_ + *φ*_−_ < 1.

In addition to the model described above (Model I), we considered an alternative Model II with a similar structure, except that the gene log RR coefficients *β*_*g*_ are not exponentiated:

(9) logit Pr⁡(Yi=1)=Xi′α+  ∑g=1NGβgG(Wg,Sig)+offseti,                         βg≥0.

To ensure that they are positive, the second level of the hierarchical model is in the following form:

(10) $$\begin{array}{c} \text{Pr}\left(\beta_{g}\right)=\{\begin{array}{cc} \varphi \left(\frac{\beta_{g}-Z_{g}^{\prime }\pi }{\sigma }\right) & \beta_{g}>0\\ \Phi \left(-\frac{Z_{g}^{\prime }\pi }{\sigma }\right) & \;\beta_{g}=0, \end{array} \end{array}$$

where *φ* denotes the probability density of normal distribution and Φ denotes the cumulative density of normal distribution. This is a proper density for *β*_*g*_, since it integrates to one. The third level of Model II remains the same as Model I. Model fitting is similar to Model I except for some details in updating *β*_*g*_s and ***π***s.

In the simulations, Model II yielded a total of 47 causal SNPs in 25 of the genes on average. Model I showed higher sensitivity and specificity for SNP selection (Table 2) than Model II based on both posterior SNP inclusion and SNP BFs. Model II showed a higher sensitivity for gene selection than Model I based on the posterior gene inclusion, but a lower specificity. In addition, Model I showed a higher sensitivity based on gene BFs.

In the application to WECARE data, Model II identified 5 SNPs in genes* MDC1*, *NBN*, and* RAD51*, with positive evidence for disease association (BF > 3). Four (rs4713354, rs2269705, rs9297757, and rs1801320) of the five selected SNPs are in common with Model I, two (rs4713354, rs9297757) are in common with Brooks et al. [25], and one (rs11620361) is not in common with previous methods. One gene (*MDC1*) was selected with positive association based on gene-level Bayes factors (BF = 6). Both the simulation study and real data application suggested that Model I performs better than Model II in terms of selecting causal variants.

We have extended the model to incorporate gene-environment (*G* × *E*) interactions with radiotherapy or radiation dose since the focus of the WECARE study is on these genes acting in response to the DSB damage induced by radiotherapy exposure. Extending the model to incorporate *G* × *E* interactions is straightforward, simply adding the main effect of *E* and an additional vector of interaction terms to the subject-level model and then putting a similar prior on the interaction coefficients. For the time being, we have treated the **β**s and *δ*s as independent, but a more appealing approach would be to treat them as having bivariate normal distributions depending on **Z** and **A**. No significant *G* × *E* interactions were found in this model (results not shown).

It remains to be seen whether this approach is scalable to GWAS data. As currently implemented with MCMC methods, the approach would not be computationally feasible, even with parallel implementations on high-performance computing environments. However, work in progress (Quintana et al. [11, 12, 22]) suggests that analytic approximations may be possible that would obviate the need for MCMC methods.

## Figures and Tables

**Table:** (a)

SNP_True_	Average counts^a^	Posterior SNP inclusion^b^			BF^c^		
		−1	0	1	>3	>20	>150
−1	17.5	24.14%	71.75%	4.11%	25.54%	17.49%	12.46%
0	348.1	3.19%	93.76%	3.05%	3.90%	0.68%	0.19%
1	18.4	3.88%	70.19%	25.94%	28.15%	19.13%	15.54%

**Table:** (b) ^ ^

Gene_True_	Average counts^d^	Posterior gene inclusion^e^		BF^f^		
		Not included	Included	>3	>20	>150
Not included	13.9	55.95%	44.05%	3.67%	0.58%	0.15%
Included	24.1	36.55%	63.45%	27.71%	20.01%	17.14%

^a^Average counts of simulated SNP inclusion indicators based on 10 × 10 replicates.

^b^Average row percentages of the distribution of posterior SNP inclusion indicators based on 10 × 10 replicates.

^c^Average row percentages of the SNP counts among the range of the indicated Bayes factors based on 10 × 10 replicates.

^d^Average counts of simulated gene inclusion indicators based on 10 × 10 replicates.

^e^Average row percentages of the distribution of posterior gene inclusions based on 10 × 10 replicates.

^f^Average row percentages of the gene counts among the range of the indicated Bayes factors based on 10 × 10 replicates.

**Table 2:** Association between selected variants in DNA-damage response genes and CBC risk in the WECARE study.

Gene	rs number	Homozygous; reference allele		Heterozygous		Homozygous; risk allele		ln⁡RR^c^		Bayes factors	
		Case (CBC)	Control (UBC)	Case (CBC)	Control (UBC)	Case (CBC)	Control (UBC)	(95% CI)	*P* value^d^	BF SNP	BF gene
*ATM *	rs1800057^a^	680	1322	28	76	0	1	−0.47 (−0.95, −0.01)	0.046	4.58	1.41
	rs4987951^a^	674	1278	34	121	0	0	−0.66 (−1.32, −0.25)	0.002	9.04	

*CHEK2 *	rs6005861^a,b^	680	1311	27	86	1	2	−0.40 (−0.85, 0.06)	0.086	7	0.36

*MDC1 *	rs4713354^a,b^	535	1116	157	267	16	16	0.47 (0.26, 0.68)	<0.001	9.72	20.71
	rs2269705^a^	589	1220	113	175	6	4	0.50 (0.25, 0.76)	<0.001	15.91	

*MRE11A *	rs13447682^a,b^	690	1343	18	54	0	2	−0.56 (−1.12, −0.01)	0.046	5.7	0.52

*NBN *	rs14448^b^	640	1215	60	171	8	13	−0.11 (−0.40, 0.18)	0.447	0.2	2.62
	rs9297757^a,b^	651	1233	148	52	5	18	−0.26 (−0.58, 0.05)	0.097	27.33	
	rs3736640^a,b^	676	1288	32	107	0	4	−0.64 (−1.27, −0.21)	0.003	4.14	

*RAD51 *	rs1801320^a^	646	1209	58	186	4	4	−0.31 (−0.62, 0.00)	0.048	21.38	3.51

^a^SNPs identified by Model I based on Bayes factors. Only those SNPs with BF exceeding 3 are listed.

^b^SNPs identified by Brooks et al. 2012 [25] based on per-allele RR. Only those SNPs with *P* value for trend <0.05 are listed.

^c^ln⁡RR: regression coefficients of each SNP from simple logistic regression, adjusted for age, menarche, menopause, family history, pregnancy, histology, treatment, the *FGFR2* GWAS-identified SNP, and deleterious variants in *ATM, BRCA1, BRCA2, CHECK2,* and offset term.

^d^ *P* values associated with Wald-z test for ln⁡RR estimates from simple logistic regression adjusted for fixed covariants listed in d.

## Appendix

### Model Fitting

At each iteration, the following updates are performed.

Selection of SNPs to include in the model involves evaluating the three posterior probabilities for *d* = {−1, 0, + 1} and selecting *W*_*s*_ with the corresponding probability

(A.1) [Wgs=d ∣ Y,S,W;  φ] ∝[Y ∣ {Gg(Wgs=d,Wg(−s),Sg),G−g};{βgsd, β−g}]  ×[Wgs=d ∣ φd,NSg],

where *β*_*gs**d*_ is a single Newton step iteration towards the maximum likelihood estimate (MLE) of *β*_*g*_ if *W*_*gs*_ were set to *d*.

Update the vector of regression coefficients **β** using a multivariate Metropolis-Hastings move with proposal **β**′ ~ *MVN*(**β**, *δ*_*β*_ **I**) and acceptance probability

(A.2) $$\begin{array}{c} \min\left\{\frac{p\left(Y\;|\;G\left(S,W\right),\beta^{\prime }\right)p\left(\beta^{\prime }\;|\;Z\pi +b,\sigma^{2}I\right)}{p\left(Y\;|\;G\left(S,W\right),\beta \right)p\left(\beta \;|\;Z\pi +b,\sigma^{2}I\right)},1\right\}. \end{array}$$

Update the vector of random effects **b** with a similar Metropolis-Hastings move with acceptance probability

(A.3) $$\begin{array}{c} \min\left\{\frac{p\left(\beta \;|\;Z\pi +b^{\prime },\sigma^{2}I\right)p\left(b^{\prime }\;|\;\tau^{2}A\right)}{p\left(\beta \;|\;Z\pi +b,\sigma^{2}I\right)p\left(b\;|\;\tau^{2}A\right)},1\right\}. \end{array}$$

Note that an alternative possibility would be to sample **β** from its marginal distribution

(A.4) [β ∣ Z,π,A,Y,S,W, σ2]∝[Y ∣ G(W,S);β] ×[β ∣ Z′π,σ2I+τ2A]

and omit the update of the **b**s.

Update the prior regression coefficients *π* by a simple linear regression and taking a multivariate normal around its MLE,

(A.5) $$\begin{array}{c} \left[\pi \;|\;\beta ,Z,b,\sigma^{2}\right]\propto \left[\beta \;|\;Z^{\prime }\pi +b,\sigma^{2}I\right]\left[\pi \;|\;0,V_{\pi }I\right]. \end{array}$$

Update the variances *σ* ^2^ and *τ* ^2^ using a Metropolis-Hastings move with proposals ln⁡(*σ*′) ~ *N*(ln⁡(*σ*),  *δ*_*σ*_) and similarly for *τ*, with acceptance probabilities

(A.6) $$\begin{array}{c} \left[\sigma ,\tau \;|\;\beta ,\;Z,\;\pi ,\;A\right]\propto \left[\beta Z^{\prime }\pi ,\sigma^{2}I+\tau^{2}A\right]\left[\sigma^{2}\right]\left[\tau^{2}\right]. \end{array}$$

As noted above, we treat the *φ*s as fixed, but these too could be given prior distributions and estimated as well.

The coefficients (*α*) of subject-level confounders are updated using single Newton-Raphson iteration towards the MLE of *α*, following a random multivariate normal update to sample the new *α*. The procedure is based on the approximation that the likelihood for *α* is quadratic with flat priors.
